# Dexamethasone-loaded keratin films for ocular surface reconstruction

**DOI:** 10.1007/s10856-021-06638-z

**Published:** 2022-03-04

**Authors:** Rebekka Schwab, Stephan Reichl

**Affiliations:** Institut für Pharmazeutische Technologie, Technische Universität Braunschweig, Braunschweig, Germany

**Keywords:** Hair keratin, Keratin film, Dexamethasone, Drug release, Ocular surface

## Abstract

Amniotic membrane (AM) is often applied as a substitute material during ocular surface reconstruction. However, since AM has several disadvantages, alternative materials must be considered for this application. Keratin films made from human hair (KFs) have previously been presented as a promising option; they exhibited suitable characteristics and satisfactory biocompatibility in an in vivo rabbit model. Nevertheless, dexamethasone (DEX) eye drops are necessary after surgery to suppress inflammation. Since eye drops must be administered frequently, this might result in poor patient compliance, and the release of DEX at the transplant site would be clinically beneficial. Therefore, we aimed to incorporate DEX into KFs without hindering the positive film characteristics. Drug-loaded KFs were generated either by suspension technique or by the addition of solubilizing agents. The resulting specimens were analyzed regarding appearance, loading capacity, transparency, mechanical characteristics, swelling behavior and in vitro release. Furthermore, biocompatibility was assessed in vitro by determining the cell viability, seeding efficiency and growth behavior of corneal epithelial cells. The amount of incorporated DEX influenced the transparency and biomechanical properties of the films, but even highly loaded films showed properties similar to those of AM. The suspension technique was identified as the best incorporation approach regarding chemical stability and prolonged DEX release. Moreover, suspended DEX in the films did not negatively impact cell seeding efficiencies, and the cell-growth behaviors on the specimens with moderate DEX loads were satisfactory. This suggest that these films could comprise a suitable alternative material with additional anti-inflammatory activity for ocular surface reconstruction.

**Figure:**
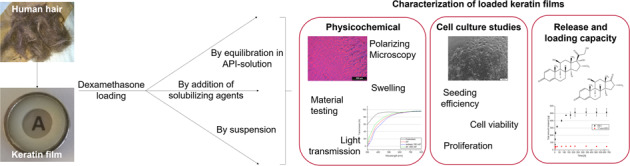
Graphical Abstract

Graphical Abstract

## Introduction

Serious ocular surface disorders, such as membrane pemphigoid, Stevens-Johnson syndrome, severe dry eye, injuries, and chronic inflammation of the eye, are major hazards to the patient’s vision and can cause corneal blindness. Since only limited pharmacological options are available to treat this clinical need, reconstruction of the ocular surface is often necessary to maintain eyesight. Preferably, human donor material in the form of corneal transplantation is utilized for this purpose [1, 2]. Nevertheless, limited supplies make it necessary to search for additional materials, and the amniotic membrane (AM) has been established as an alternative for the treatment of corneal epithelial defects or replacement of the conjunctiva [3]. It is also an advantage that limbal stem cell deficiency can be treated by expanding limbal epithelial stem cells on the membrane before transplantation [4–7]. The amnion membrane is the inner layer of the maternal placenta and amniotic sac that closely covers the fetus during formation in the womb. The placenta is donated as living tissue donation after a planned cesarean section. Amnion membranes are used in medicine due to their many beneficial characteristics, such as outstanding wound healing properties, antiangiogenic features and anti-inflammatory capabilities [3, 6, 8]. However, since the amnion membrane is a human material, some challenges must be considered to enable application of such membranes. For example, the supply is strictly regulated and restricted, and the use of such membranes risks disease transmission. Furthermore, the material transparency and biomechanical strength are limited [8–11].

A possible alternative for amnion membranes in ocular surface reconstruction is keratin films (KFs), which are prepared from human hair. Keratins are structural proteins found in the hard or filamentous structures, such as feathers, hoofs, wool, hair or nails, of higher vertebrates. These cysteine-rich proteins form disulfide bonds and therefore are quite stable and water-insoluble [12–15]. Since the extraction of keratin is complex and time consuming, only a few attempts have been made to process these proteins into fabrics for regenerative medical use [13, 14]. However, as shown previously, films made from human hair keratin have been characterized to have excellent transparency, good biomechanical characteristics and high cell-attachment efficacy [16]. Furthermore, it is easier to standardize such films, as compared to AMs, and KFs have displayed satisfactory biocompatibilities in an in vivo rabbit model [17]. Nevertheless, the animals in this study showed some signs of inflammation after implantation, and anti-inflammatory eye drops were necessary to prevent rapid implant degradation [17]. Therapeutic regimens for topically applied corticosteroids are rigid and often comprise frequent drug administration [18, 19]. This might cause limited compliance and could be a challenge for clinical feasibility since patient adherence to the therapy is essential to avoid rapid degradation of the transplanted material [17]. For this reason, this study aims to incorporate the anti-inflammatory corticosteroid dexamethasone (DEX) as an active pharmaceutical ingredient (API) into established KFs. Topical corticosteroids such as DEX have been frequently applied as a medicinal option for the prevention of ocular inflammation, neovascularization and scarring [18–23]. Corticosteroids hinder inflammatory processes by reducing proinflammatory cytokines and VEGF expressions [19, 24, 25] and prevent scarring by suppressing cell division [22, 26].

Different approaches for DEX incorporation were explored, and their impacts on film characteristics were investigated. Direct DEX incorporation was realized through the introduction of an API during the film-manufacturing process. Subsequent DEX loading was achieved by soaking the films in an API solution. The effects of drug incorporation on the appearance/transparency, mechanical behavior, swelling, and biocompatibility were monitored. The in vitro release from DEX-loaded films was analyzed to estimate the potential of the materials to release drugs directly at the transplant site. Thus, frequent drug administration after surgery could be avoided, and some essential clinical problems that occur during ocular surface reconstruction, namely, inflammation, neovascularization, and scarring, could be solved.

## Material and methods

### Materials

DEX was kindly provided by Ursapharm (Saarbrücken, Germany). Urea, thiourea, glycerol, sodium hydroxide, methanol, sodium dodecyl sulfate (SDS) and Triton-X-100 were purchased from Carl Roth (Karlsruhe, Germany). Polysorbate 80 and 2-amino-2-hydroxymethylpropane-1,3-diol (Tris) were obtained from Caesar & Loretz (Hilden, Germany). Acetonitrile, 2-mercaptoethanol, polyoxyl 40 stearate and Ellman’s reagent (5,50-dithiobis-(2-nitrobenzoic acid)), fetal calf serum (FCS), human insulin, dimethyl sulfoxide (DMSO), 3-(4,5-dimethyl-2-thiazolyl)-2,5-diphenyl-2H-tetrazolium bromide (MTT), hydrochloric acid, isopropanol and an antibiotic antimycotic solution were purchased from Sigma-Aldrich (St. Louis, U.S.). 17-oxo-DEX was obtained from Toronto Research Chemicals (North York, Canada). Phosphate-buffered saline (PBS) and ethylenediaminetetraacetic acid (EDTA) were purchased from MP Biomedicals (Solon, U.S.). Trypsin-EDTA solution from Gibco (Paisley, UK) was used. Dulbecco’s modified Eagle’s medium (DMEM), Ham’s F12 medium and human epidermal growth factor (EGF) were purchased from PAN-Biotech (Aidenbach, Germany). Ethanol was ordered from Fisher Scientific (Loughborough, UK). A Spectra/Por^®^ 1 dialysis membrane (molecular weight cut-off (MWCO) 6–8 kDa) was purchased from Spectrum Laboratories (Rancho Dominguez, U.S.), and polystyrene 96- and 24-well cell culture plates were obtained from TPP (Trasadingen, Switzerland). Vivaspin^®^ 20 (MWCO 5 kDa) were obtained from Sartorius (Goettingen, Germany), and polyethylene terephthalate (PET) foil was acquired from LTS Lohmann (Andernach, Germany). PureVision^®^ soft contact lenses were purchased from Bausch & Lomb (Rochester, U.S.). Double-distilled water was used. Krebs-Ringer buffer (KRB) was prepared by dissolving 6.80 g of NaCl (Carl Roth), 0.40 g of KCl (Thermo Fisher Scientific, Waltham, U.S.), 0.14 g of NaH_2_PO_4_·H_2_O (Merck, Darmstadt, Germany), 2.10 g of NaHCO_3_ (Carl Roth), 3.58 g of 2-[4-(2-hydroxyethyl)piperazin-1-yl]ethanesulfonic acid (HEPES; Carl Roth), 1.10 g of D-glucose monohydrate (Carl Roth), 0.20 g of MgSO_4_·7 H_2_O (Thermo Fisher Scientific) and 0.26 g of CaCl_2_·2 H_2_O (Carl Roth) in 1000 mL of double-distilled water.

### Keratin extraction

Blond hair was obtained from a local hairdresser. The hair was washed with 0.5% SDS in water and rinsed thoroughly with water before air-drying. Keratin extraction was performed according to the Shindai method [27]. Shindai solution was prepared by dissolving 25 mM Tris, 2.6 mM thiourea, 5 M urea and 5% 2-mercaptoethanol in water and adjusting the pH to 8.5. In brief, Shindai solution (400 mL) was added to the hair (20 g) as an extraction medium for 72 h at 50 °C. The batch was stirred gently. Afterwards, the blend was centrifuged at 5000 rpm for 30 min before the supernatant (the Shindai extract) was filtered and stored at −20 °C until needed for experiments.

To remove cytotoxic agents (e.g. thiourea and 2-mercaptoethanol) from the extract, it was dialyzed extensively against water or 0.25 M sodium hydroxide [16, 28]. For aqueous dialysis, 100 mL of extract was dialyzed against 5 L of water using an MWCO 6–8 kDa cellulose dialysis tube (Spectra/Por^®^) at room temperature for 6 days. The dialysis fluid was changed daily. The absence of mercaptoethanol in the dialysis medium was verified via Ellmann’s reagent [29] before the aqueous keratin dialysate was centrifuged for 30 min at 10,000 rpm for purification. For alkaline dialysis, 40 mL of extract was dialyzed against 2 L of 0.25 M sodium hydroxide, as described above, at 4 °C. Both batches were stirred mildly during dialysis. After dialysis, the alkaline dialysate was concentrated using MWCO 5 kDa Vivaspin^®^ 20 concentrators. The supernatant was diluted repeatedly with 0.05 µM sodium hydroxide to produce the final alkaline dialysate for film preparation.

The protein amount was monitored via Bradford assay as described previously [30]. Ultraviolet (UV) absorption was measured with an Infinite M Plex multiplate reader (Tecan, Männedorf, Switzerland).

### Keratin film manufacturing

KF manufacturing was performed as described previously [16, 17, 31]. In brief, aqueous dialysate was mixed with alkaline dialysate at a ratio of 90 to 10. As a softening agent, 1% glycerol was added. Then, 1600 µL of the blend was cast into Teflon molds with inner diameters of 20 mm, which were fixed on hydrophobic coated PET sheets. The dialysate was air-dried for 48 h to generate plain and clear KFs. Upon curing for 2 h at 110 °C, the films were transformed to water-insoluble, biomechanically stable specimens. For in vitro release testing, films were correspondingly prepared from a 700 µL dialysate mixture.

Microscopic examination of the resulting films was conducted with a polarizing microscope (DMLM, Leica, Wetzlar, Germany) equipped with a camera (MC 170 HD, Leica).

### Drug loading of keratin films

#### Direct incorporation of DEX

First, DEX was incorporated into KFs by dissolving the drug in the dialysate mixture. An excess amount of DEX (~10 mg) was added to 10 mL of the blend and equilibrated at 20 °C on an orbital shaker (100 rpm) for 72 h. To remove any undissolved DEX, the fluid was filtered with 0.45 µm Rotilabo^®^ RC syringe filters (Carl Roth). To obtain directly loaded DEX-KF, the saturated DEX dialysate was processed as described above (Section “Keratin film manufacturing”).

The DEX solubility in the dialysate mixture was analyzed in agitated glass flasks. An excess amount of DEX (~10 mg) was added to 10 mL of dialysate mixture and equilibrated at 20 °C on an orbital shaker (100 rpm) for 72 h. Samples were withdrawn from the bulk and filtered (0.45 µm Rotilabo^®^ RC syringe filters, Carl Roth). The DEX was quantified based on high-performance liquid chromatography (HPLC) after dilution, as described below (Section “In vitro release study”). This examination was performed in triplicate.

Higher amounts of DEX were incorporated by the addition of solubilizing agents and the formation of mixed micelles via the rotary evaporation method, as reported previously [32]. In brief, a solution of DEX (10 mg) and polyoxyl 40 stearate (P40S, 420 mg) in ethanol (5 mL) was prepared. The organic phase was evaporated under reduced pressure and rotation at 50 °C, resulting in a thin film of the drug and P40S in the round bottom flask. Polysorbate 80 (PS80, 180 mg) was dissolved in 10 mL of dialysate mixture, and the solution was added to the flask for the formation of DEX-mixed nanomicelles. The solution was filtered with polyethersulfone (PES) syringe filters (0.45 µm, Filtropur, Sarstedt, Nümbrecht, Germany) and processed into KFs (MKF 1000). To obtain films with lower drug loads, the micellar dialysate was diluted with a dialysate mixture to the final DEX concentrations of 500 or 250 µg/mL (MKF 500 and MKF 250).

Another approach for achieving higher drug loads was implemented by suspension of fixed DEX amounts (250, 500, or 1000 µg/mL) within the dialysate mixture. The dialysate was incubated with the drug at 20 °C on an orbital shaker (100 rpm) for 72 h to guarantee the mixture was at equilibrium. Afterwards, films were processed as discussed before (Section “Keratin film manufacturing”) to obtain SKF 250, SKF 500, and SKF 1000.

#### Subsequent incorporation of DEX

Round specimens were punched out from unloaded KFs at diameters of 18 mm, and 2 mL of a saturated DEX-PBS solution was subsequently added to generate loaded DEX-KFs. The DEX solubility in PBS was analyzed in agitated glass flasks, as discussed above, to examine DEX solubility in dialysate mixtures (see Section “Direct incorporation of DEX”). The films were incubated at 20 °C for various time intervals. After each of these predetermined time intervals, the KFs were withdrawn, and excess fluid was wiped off with tissue paper. To analyze the realized drug load, films were dried for 48 h and extracted for 24 h with 2 mL methanol during stirring (200 rpm). Drug quantification was performed by HPLC as described below (Section “In vitro release study”). The drug-loading capacity was determined in triplicate.

### In vitro characterization of drug-loaded keratin films

#### Transparency measurement

**Fig. 1:**
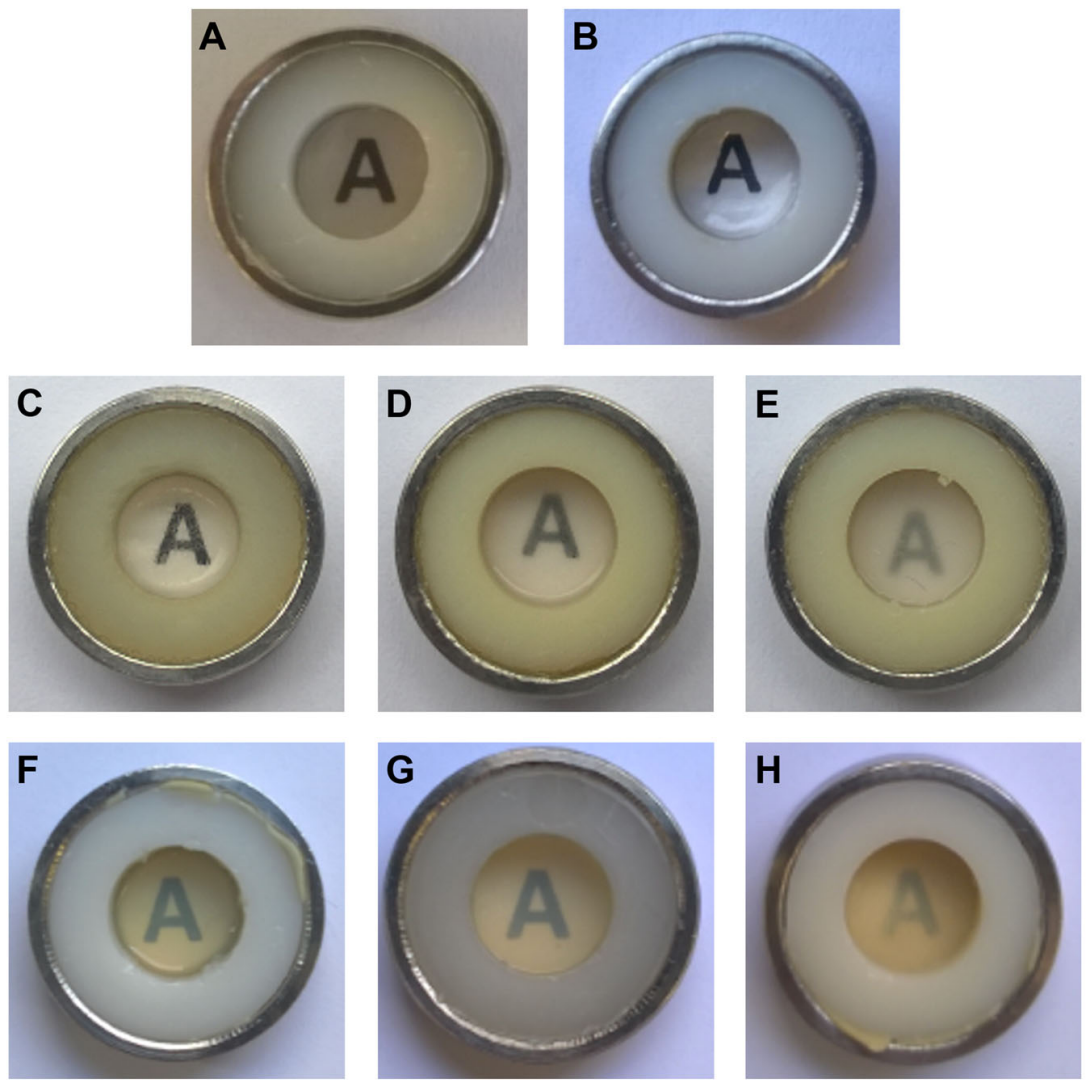
Macroscopic transparencies of **A**, **B** an unloaded keratin film, a film fabricated from DEX saturated dialysate (KF = **A** and dir. DEX-KF = **B**), **C**, **D**, **E** films fabricated from different DEX dialysate suspensions (SKF 250 = **C**, SKF 500 = **D**, and SKF 1000 = **E**), and **F**, **G**, **H** films manufactured with different amounts of solubilized DEX (MKF 250 = **F**, MKF 500 = **G**, and MKF 1000 = **H**) as illustrated by the legibility of the letter beneath the substrates

To secure the films and generate a standardized area for further characterization, KFs were punched out (diameter 11 mm) and fitted in custom-constructed two-ring arrangements as described previously [16] and displayed in Fig. 1. With this apparatus, a free film surface of 0.332 cm^2^ was obtained. To evaluate the macroscopic appearance and transparency, each film was placed on a printed “A” (Calibri font, size 14), and the legibility of the letter was observed through the film.

The light transmission was calculated based on extinction, which was measured in the wavelength range of 300–800 nm with a resolution of 1 nm and blank correction. Measurements were performed on three films with a spectrophotometer (Infinite M Plex, Tecan). These films were equilibrated in water for 15 min and fitted into the described two-ring arrangements; then, they were positioned in a 24-well plate filled with 1 mL of water per well. For comparison, three therapeutic soft contact lenses (PureVision^®^ BC 8.6 PWR 0.00) were measured under the same conditions.

#### Film-swelling behavior

The films were incubated in 5 mL of double-distilled water for 24 h at 20 °C. After each predetermined time interval, the film diameter was measured with a measuring slide and compared to the diameter of the dry film. The increases in KF size were calculated as percentages. The measurements were conducted on six films.

#### Material testing

Mechanical characteristics were evaluated by means of a zwicki-Line Z 0.5 static material testing machine (Zwick, Ulm, Germany) with a 10 N load cell and testXpert^®^ II software. The films were equilibrated in double-distilled water for 15 min, and dumbbell-shaped specimens (35 mm length) were punched out using a film-cutting device (Zwick). A test speed of 1 mm/min was chosen for E-modulus testing, and a speed of 5 mm/min was set for tensile strength evaluation. The obtained stress-strain curves (provided as supplementary data) were utilized to calculate the E-modulus (EM, Young’s modulus) and ultimate tensile strength (US). The EM describes the stiffness or deformability of an isotropic elastic material, whereas US represents the maximum stress that a film can withstand before it is torn apart.

#### In vitro release study

Two milliliters of preheated PBS (37 °C) was added to the film substrates for in vitro release testing. Films were incubated under gentle agitation on an orbital shaker (150 rpm) at 37 °C. After each predetermined time interval, 1 mL of PBS was withdrawn from the vials and replaced with fresh fluid. This investigation was conducted in triplicate. DEX quantification in the samples was performed based on HPLC analysis using a Waters HPLC equipped with a pump and controller (Model 600), an autosampler (Model 717plus) and a UV absorbance detector (Model 486) (Waters, Milford, U.S.). The mobile phase consisted of water and acetonitrile (60:40 (v/v)), and an Equisil ODS C18 column with a precolumn (5 µm C18, 125 × 4 mm; Techlab, Braunschweig, Germany) was chosen as the stationary phase. The flow rate was 1 mL/min, and the detection wavelength was set at 238 nm. Twenty microliters of the samples were injected, and the DEX retention time was found to be ~3 min. Calibration was performed in the range from 0.2 to 20 µg/mL (*r*^2^ > 0.999). Furthermore, the DEX degradation product 17-oxo-dexamethasone (17-oxo-DEX) was quantified. The peak-retention time for this compound was ~5 min, and calibration was conducted in the span between 0.2 and 26 µg/mL (*r*^2^ > 0.999). Peak integration and evaluation were carried out with Clarity^TM^ 8.0 software (DataApex, Prague, Czech Republic).

#### Cell culture

HCE-T cells were chosen for investigation of cell growth and seeding on DEX-loaded KFs. HCE-T cells belong to a thoroughly characterized corneal epithelial cell line that emerged from infection of human corneal epithelial cells with a recombinant SV40-adenovirus vector [33]. The cell line was obtained from RIKEN Cell Bank (Tsukuba, Japan). For cultivation, cells were incubated in a mixture comprising equal parts of DMEM and Ham’s F12 medium, containing 5% FCS, 5 µg/mL insulin, 10 ng/mL EGF, 0.5% DMSO and 1% antibiotic/antimycotic solution. The medium was exchanged three times per week, and incubation was performed in a humidified atmosphere at 37 °C with 5% supplemented CO_2_. The absence of mycoplasma was monitored by PCR (test kit by PromoCell, Heidelberg, Germany). Previous studies used an intensive washing protocol to prepare films for experiments [16, 17, 31]. According to the established protocol (protocol A), KFs were equilibrated in water with 1% antibiotic/antimycotic solution for 7 days, and the fluent solution was exchanged twice daily. Two alternative shortened washing protocols (protocols B and C) were investigated to minimize drug loss. Films were washed for 90 min with fluid replacement after 30 and 60 min (protocol B) or for 30 min with no replacement at all (protocol C). To prove the suitability of these shortened washing periods, the seeding efficiency on regularly prepared KFs (protocol A) was compared to the performance on alternatively prepared KFs (protocols B and C) as described below (Section “Seeding efficiency”). After washing, films were secured in the two-ring apparatus, as described for transparency measurements (Section “Transparency measurement”). Before seeding cells onto the films, the KF was stored in the medium for 15 min. In a second step, the films from saturated DEX dialysate and the films with low and moderate amounts of suspended DEX were investigated. To maintain as much drug content in the films as possible, washing protocol C was used for film preparation in these experiments (see Sections “Seeding efficiency” and “Characterization of cell proliferation”).

#### Viability testing

An MTT assay was performed to assess cell viability. In a clear 96-well cell culture plate, 18,000 HCE-T cells were seeded per well and incubated for 48 h. Afterwards, the medium was removed, and the cells were washed thoroughly with KRB. Saturated DEX and 17-oxo-DEX solutions were prepared as samples in KRB. Furthermore, unloaded KFs and KFs with directly incorporated DEX were incubated with KRB for 30 min, and the fluid was replaced after 15 min as described above for release testing (Section “In vitro release study”). The obtained release media were also utilized as test solutions. Samples were pipetted onto the cells and incubated for 30 or 240 min. Then, test solutions were withdrawn from the cells, and a 0.05% MTT solution was placed on the cell layers. After an incubation period of 3 h, the solution was replaced by a fluid for cell lysis. The lysis solution consisted of 0.27 g of SDS, 0.36 g of hydrochloric acid, 8.82 g of water, and 90.55 g of isopropanol. After 30 min, UV absorption at 570 nm was quantified by means of a multiplate reader (Infinite M Plex, Tecan). KRB was used as a negative control (untreated cells), and 1% Triton-X-100 solution was placed on the cells as a positive reference. The results were related to the untreated control and calculated as percentages.

#### Seeding efficiency

To investigate the seeding efficiency, 50,000 cells per film were seeded within the ring arrangements described above (see Section “Transparency measurement”); this is equivalent to a density of 150,000 cells per cm^2^. The cells were incubated for 8 h before the films were thoroughly rinsed with PBS. The cells were detached with trypsin/EDTA and counted with a Z2 Coulter counter (Beckman Coulter, Krefeld, Germany). These measurements were performed in quadruplicate or octuplicate. To determine the seeding efficiency, the ratio of attached cells after 8 h to the originally seeded cells was calculated.

#### Characterization of cell proliferation

To characterize the proliferation of HCE-T cells on the film specimens, the established ring devices (Section “Transparency measurement”) were used once more. A total of 30,000 cells were seeded onto each of the films to realize a cell density of 90,000 cells per cm^2^. Afterwards, the cells on the specimens were allowed to grow for a period of 14 days. Proliferation was monitored microscopically with a phase contrast microscope (Olympus IX 50, Olympus, Hamburg, Germany), and micrographs were taken with a camera (Olympus XC 30, Olympus). After predetermined time intervals, the cells were counted after they were rinsed with PBS and subsequently detached from the films with trypsin/EDTA (see Section “Seeding efficiency”). The cell number was expressed as the mean of four or five wells, and a sigmoid curve was fitted to the data points after semilog-plotting against the culture time. To determine the population doubling time (PDT), a straight line was drawn through the almost-linear part in the middle of the sigmoid fit. By extrapolating this line and determining its intersection with the initial cell number, the lag phase was assessed. The cell number that corresponded to the plateau phase at the end of the proliferation study was assumed to be the saturation density. Data evaluation was performed using OriginPro^®^ software (OriginLab, Northampton, U.S.).

### Statistical evaluation

All results are shown as the mean value ± standard deviation. For statistical analysis, IBM SPSS^®^ Statistics 26 software (Armonk, U.S.) was utilized to perform one-way analysis of variance (ANOVA) with Bonferroni or Games-Howell post-hoc tests for homogenous or inhomogeneous variances, respectively. For *P* < 0.05, statistical significance was assumed.

## Results

### Film characteristics

In Fig. 1, the transparencies of KFs with different loading properties are compared. As indicated by the clarity and legibility of the letter beneath the films, the KF and directly loaded DEX-KF were characterized by a high transparency. The incorporation of DEX within the saturation solubility did not limit the transparency (Fig. 1B). In contrast, films fabricated from keratin dialysates with solubilized or suspended API showed lower transparency levels (Fig. 1C–H). With increasing drug load, the transparency decreased even more. In general, the transparencies of loaded KFs with equal amounts of DEX were higher for KFs manufactured from dialysate suspensions than for those manufactured from dialysates with solubilized DEX. The films with low or moderate amounts of suspended DEX (SKF 250 and SKF 500) showed a satisfactory recognizability of the underlying “A” (Fig. 1C, D).

**Fig. 2:**
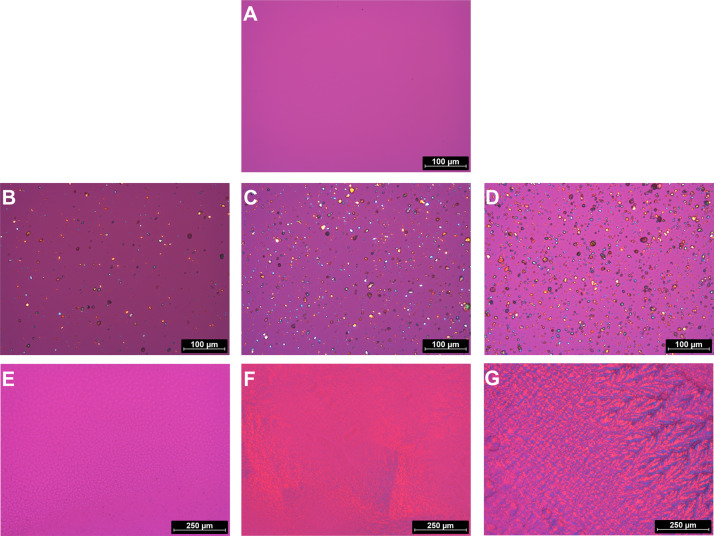
Polarizing micrographs of **A** a film fabricated from DEX saturated dialysate (dir. DEX-KF = **A**), **B**, **C**, **D** films fabricated from different DEX dialysate suspensions (SKF 250 = **B**, SKF 500 = **C**, and SKF 1000 = **D**), and **E**, **F**, **G** films manufactured with different amounts of solubilized DEX (MKF 250 = **E**, MKF 500 = **F**, and MKF 1000 = **G**)

To evaluate the presence of drug crystals within the films, polarized micrographs were taken, as displayed in Fig. 2. No drug crystals were observed in a KF from saturated dialysate (Fig. 2A); this indicates that the API did not recrystallize while the film was drying but rather appeared to be molecularly dispersed. As expected, much crystalline drug substance was detected in films with suspended DEX (Fig. 2B–D). The crystals were uniform in appearance and displayed a homogenous distribution within the films. No agglomerates were present. The films fabricated from dialysates with solubilized DEX also showed no DEX crystals (Fig. 2E–G). Films with low amounts of incorporated solubilizing agents displayed minor deviations compared to the uniform appearances of KFs and DEX-KFs. MKFs with a higher content of surfactant exhibited more diversified structures; these findings are consistent with the macroscopic evaluation results.

**Fig. 3:**
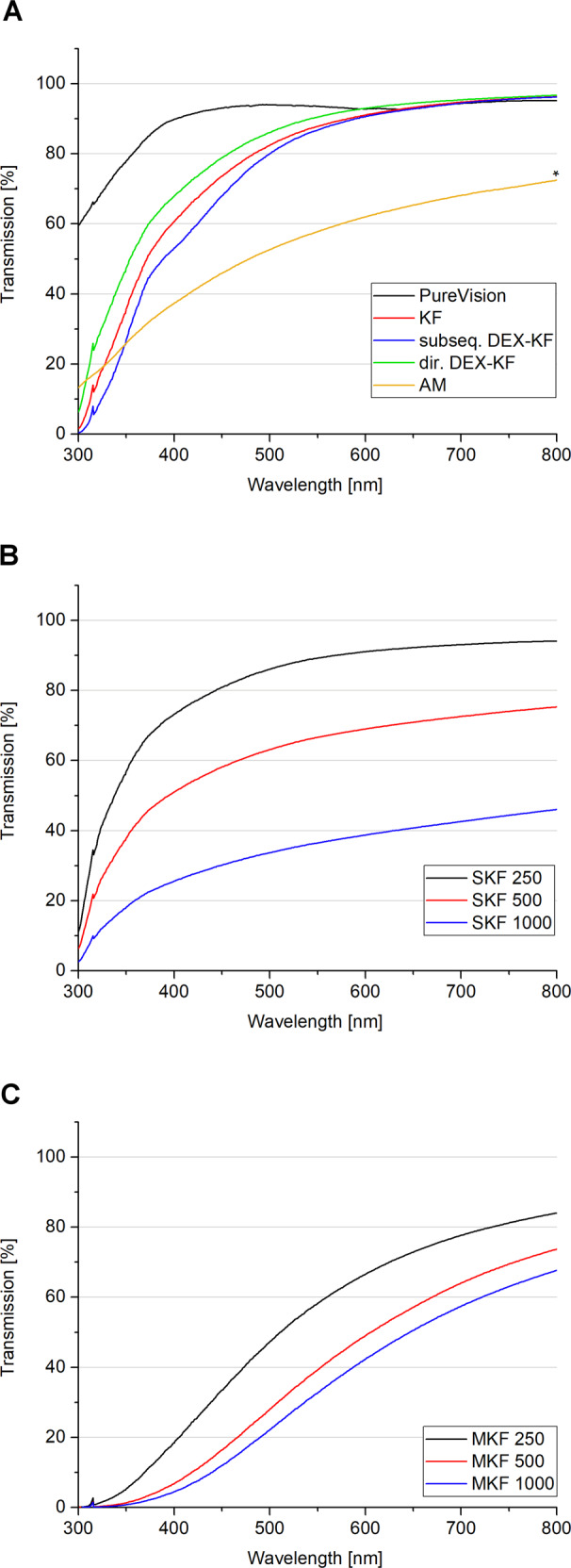
Light-transmission spectra of **A** contact lenses and unloaded keratin films, directly as well as subsequently loaded DEX keratin films and amniotic membrane, **B** films fabricated from different DEX dialysate suspensions, and **C** films manufactured with different amounts of solubilized DEX, mean, *n* = 3;* from ref. [16]

Optical findings were confirmed by light-transmission measurements, the results of which are displayed in Fig. 3. The contact lenses, which were used as a positive control, showed the highest transmission level with values of nearly 100% in the wavelength range of visible light (400–800 nm). The light transmissions of unloaded KFs were moderate to high, with values of 61% at 400 nm and 96% at 800 nm (Fig. 3A). Subsequently loaded DEX-KFs displayed light-transmission values of 53% at 400 nm and 96% at 800 nm (Fig. 3A). These results suggest that the effect of subsequent incorporation on light transmission is negligible. Direct incorporation of DEX by the saturation technique also did not show a negative impact on light transmission. In fact, such films even showed a tendency towards slightly higher light transmissions, with values of 68% at 400 nm and 97% at 800 nm (Fig. 3A). Upon incorporation of higher DEX amounts into the films, the light transmission decreased; this decrease showed an inverse correlation to the amount of incorporated DEX. For KFs manufactured from lower concentrated dialysate suspensions (SKF 250), light-transmission values of 73% at 400 nm and 94% at 800 nm were detected, and SKF 1000 transmitted only 26% at 400 nm and 46% at 800 nm (Fig. 3B). The light transmission was even more impaired when higher amounts of DEX were incorporated into KFs by the addition of solubilizing agents (Fig. 3C). In this case, especially in the lower wavelength range, less light was transmitted through the films. For MKF 250, we measured light-transmission values of 18% at 400 nm and 84% at 800 nm. The light transmissions of MKF 1000 equaled 4% at 400 nm and 68% at 800 nm. In a previous study, the light-transmission values of AM were measured to be 34% at 400 nm and 70% at 800 nm (Fig. 3A) [16], and thus, such values are within the range of values for SKF 500. The light transmission of SKF 250, which was found to be as good as that of unloaded KFs, was even superior to that of AM.

**Fig. 4:**
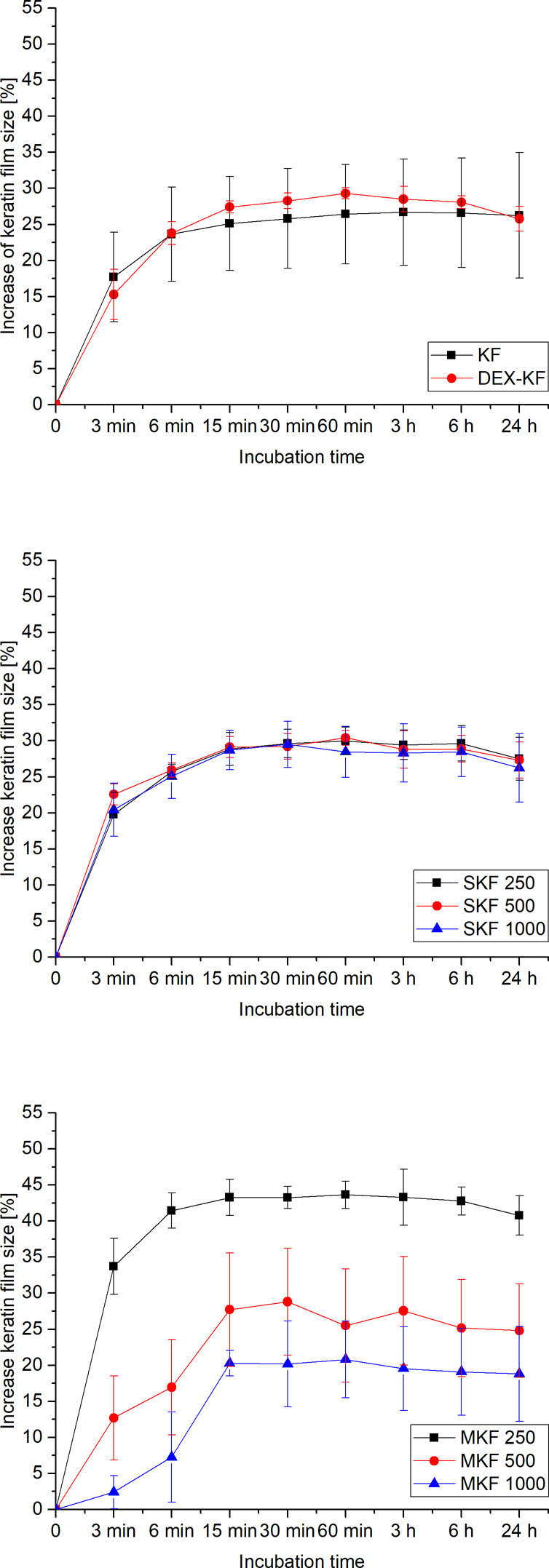
Swelling behavior of films fabricated from DEX saturated dialysate, films fabricated from different DEX dialysate suspensions and films manufactured with different amounts of solubilized DEX, as described by the increase in film diameter within an equilibration period of 24 h, mean ± SD, *n* = 6

The swelling of films during equilibration in water was studied over a period of 24 h. Figure 4 illustrates how the sizes of films loaded by different incorporation approaches and with varying drug amounts increased in relation to their original diameters. KFs displayed a maximum size expansion of 27% after 3 h of equilibration. Most of this increase was completed after only 6 min of incubation, when film swelling was measured to be 24%. Similar behavior was observed for directly loaded films fabricated from saturated DEX dialysate mixtures, in which the swelling equaled 26% after 6 min of equilibration, and a maximum increase of 30% was reached after 1 h. Augmented drug loading by suspension had no effect on swelling behavior. In contrast, the addition of solubilizing agents drastically changed the film-swelling behavior. While large amounts of incorporated PS80 and P40S in MKF 1000 led to a less pronounced swelling of only 21% after 1 h, KFs with smaller quantities of incorporated amphiphilic molecules showed distinct swelling increases. MKF 250 showed a maximum film expansion of 44% after 1 h.

**Fig. 5:**
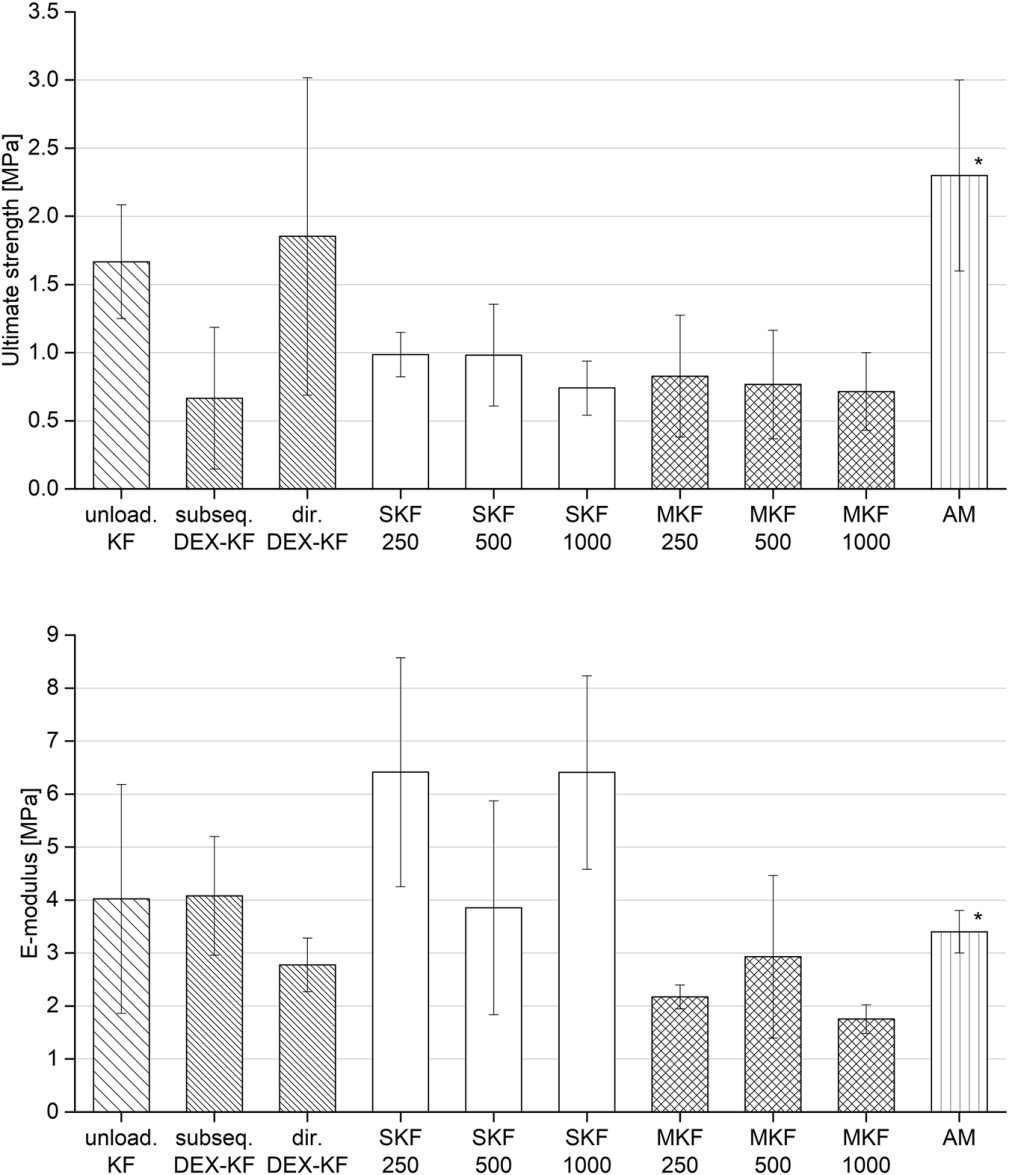
Mechanical testing results (US and EM) for unloaded keratin films (KFs) as well as for subsequently loaded films (subseq. DEX-KFs), films fabricated from DEX saturated dialysate (dir. DEX-KFs), films fabricated from different DEX dialysate suspensions (SKF 250, SKF 500, and SKF 1000), films manufactured with different amounts of solubilized DEX (MKF 250, MKF 500, and MKF 1000) and amniotic membrane, mean ± SD, *n* = 6–10;* from ref. [16]

The impacts of direct and subsequent DEX incorporation on KF mechanical characteristics is displayed in Fig. 5. The EM and US were evaluated to characterize the biomechanical properties of the specimens. The KFs displayed an US of 1.7 MPa and an EM of 4.0 MPa. Neither direct nor subsequent DEX incorporation had a major influence on the EM values (*P* > 0.05). The US slightly decreased to 0.7 MPa after subsequent KF loading (*P* < 0.05), while no such effect was detected after direct KF loading within the saturation concentration (*P* > 0.05). Loading KFs with higher amounts of DEX, either by the addition of solubilizing agents or by suspension of the API, showed a trend towards a slightly reduced US level (*P* < 0.05). The lowest US values were detected for SKF 1000 (0.74 MPa) and MKF 1000 (0.71 MPa). No distinct changes were registered concerning EM values of the SKF and MKF in comparison to the plain KF (*P* > 0.05). For human AM, an EM of 3.4 MPa and a US of 2.3 MPa were measured in a previous study [16]. Compared to these values, the DEX-KF exhibited biomechanical properties within the AM range.

### Investigation of drug load and in vitro release

To investigate the drug loading capacity of directly loaded KFs, the saturation concentration of DEX in the dialysate mixture was analyzed. This DEX concentration was measured as 112.76 ± 1.28 µg/mL. Since 1.6 mL was cast into Teflon rings, the final drug load equaled 180.42 µg for each directly loaded DEX-KF. The DEX concentration in the saturated PBS solution was 69.35 ± 1.42 µg/mL and thus was remarkably lower than that in the saturated dialysate mixture.

**Fig. 6:**
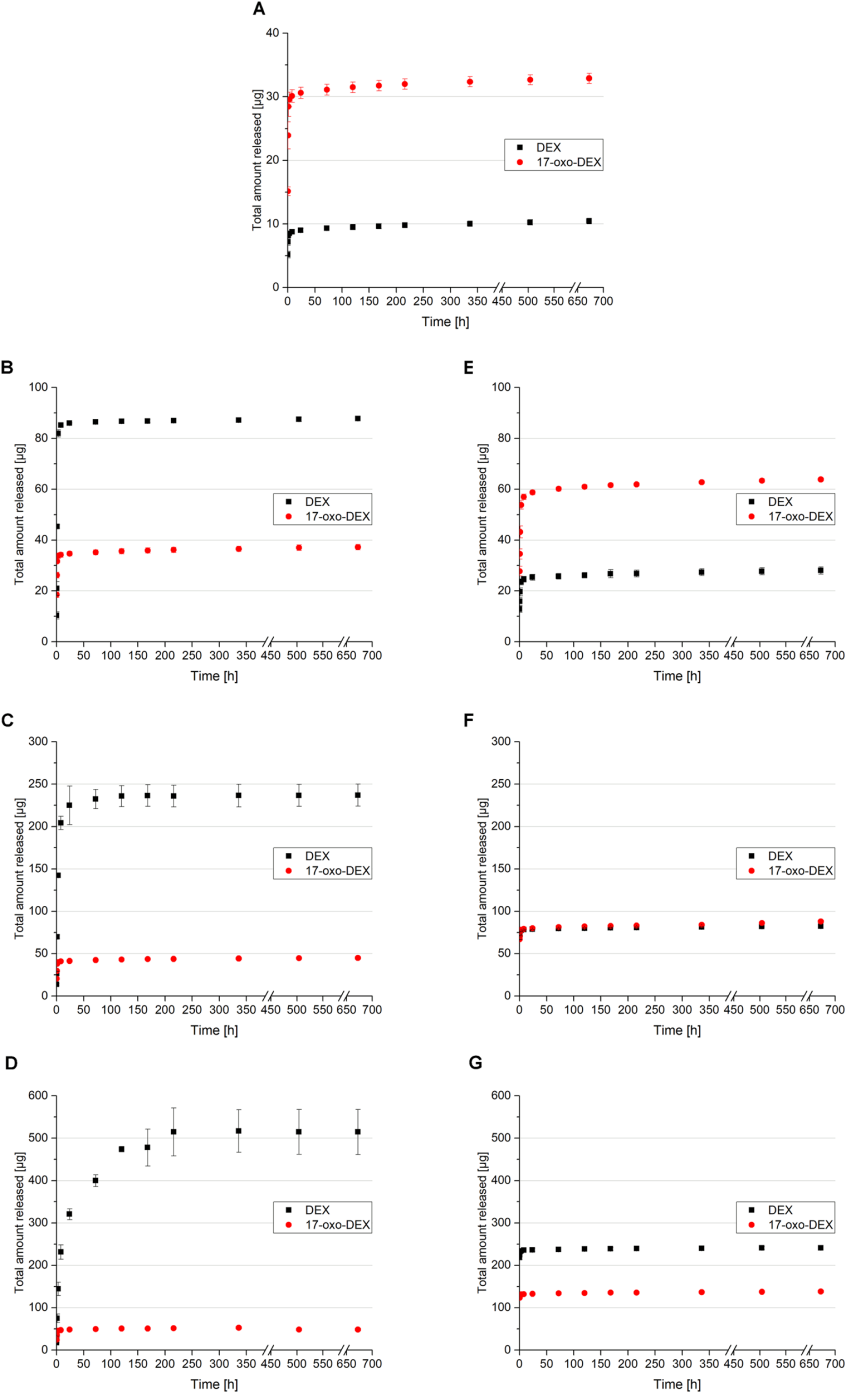
Amounts of DEX and 17-oxo-DEX released from **A** films fabricated from saturated DEX-dialysate (DEX-KF = **A**), **B**, **C**, **D** films fabricated from different DEX dialysate suspensions (SKF 250 = **B**, SKF 500 = **C**, and SKF 1000 = **D**) and **E**, **F**, **G** films manufactured with different amounts of solubilized DEX (MKF 250 = **E**, MKF 500 = **F**, and MKF 1000 = **G**) over 4 weeks, mean ± SD, *n* = 3

The drug release behaviors from KFs directly loaded by saturation, suspension or solubilization were monitored over 4 weeks. In Fig. 6, the release profiles are displayed. In addition to DEX, a notable amount of 17-oxo-DEX was released from the directly loaded films. 17-oxo-DEX is a degradation product of DEX, which results from the oxidation of the original compound. The degradation of the solved API was intensified by an alkaline environment and elevated temperatures, as observed during film curing. Abnormally, only a minor degradation of DEX into 17-oxo-DEX (<1%) was captured in the dialysate mixture at room temperature. Films that were manufactured from saturated dialysate mixtures showed pronounced burst release characteristics. Most of the API and its degradation product were released after only 24 h. Significantly more 17-oxo-DEX was released from the films than DEX; while approximately only 10 µg of DEX was released, over three times more 17-oxo-DEX was detected. Films with low amounts of incorporated solubilizing agents (MKF 250) released more 17-oxo-DEX than DEX. Due to their higher concentrations of the dissolved drug, even more 17-oxo-DEX formed in these films. The quantities of DEX and 17-oxo-DEX released from MKF 500 were equivalent, while more DEX than 17-oxo-DEX was released from films with high amounts of incorporated solubilizers (MKF 1000). Due to higher dissolved drug concentrations, more 17-oxo-DEX formed in these films, too. Furthermore, the burst release characteristics of the films with solubilizing agents were even more pronounced. Approximately 90% of the finally released API was liberated from the films within 8 h. The films that included suspended DEX released more DEX than 17-oxo-DEX. Although films with low quantities of suspended DEX (SKF 250) released more than twice as much DEX than 17-oxo-DEX, the amount of DEX released from films with moderate amounts of the suspended drug (SKF 500) was more than fivefold larger than the amount of 17-oxo-DEX released. The ratio between the drug and degradation product increased further to almost 10 times as much released from the SKF 1000. This suggests that DEX decomposition is limited by the total amount of dissolved API. In addition, prolonged drug release was detected in films with suspended DEX. The more suspended DEX, the more prominent the extension in the API liberation time. Thirty-eight percent of the finally released DEX was liberated from the films with high amounts of suspended DEX after more than 24 h. After 120 h, more than 8% of the final released drug amount had not yet been released from the films. As the DEX molecules slowly dissolved from the suspended crystals, the extension of the prolonged release was closely linked to the solubility of the active ingredient. The uniformity of the DEX crystals, as observed microscopically (see Section “Film characteristics”), is of great importance to the predictability and reproducibility of drug release profiles.

**Fig. 7:**
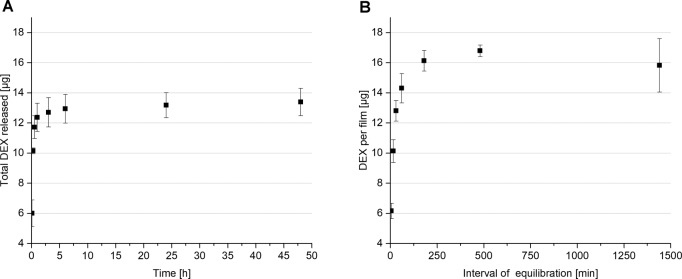
**A** DEX release from subsequently loaded DEX-KFs over 48 h and **B** drug load per keratin film after subsequent incorporation of DEX, mean ± SD, *n* = 3

To examine the subsequent incorporation approach, the influence of the loading interval on the final drug load of subsequently loaded DEX-KFs was assessed first. Thus, the films were equilibrated in a saturated DEX-PBS solution for various time periods, and the drug loads were determined upon extraction. As shown in Fig. 7B, longer loading intervals resulted in higher drug loads. The highest subsequent drug load of 16.79 ± 0.39 µg per film was achieved after 480 min of equilibration. In contrast, it did not seem useful to further extend the loading period because, after 24 h of equilibration, the detected subsequent drug load dropped to 15.82 ± 1.77 µg. Hence, 480 min was established as a standard loading interval for further investigations. In the course of these experiments, the drug release behaviors of subsequently loaded films were studied; these results are presented in Fig. 7A. DEX was rapidly and fully released from the films within 48 h. Afterwards, no further drug release was detected. This result suggests that DEX is loosely attached to the film surface by nonspecific hydrophobic and hydrophilic interactions. These weak linkage characteristics do not enable extended release. Apart from this, the realizable drug load is rather low, so subsequent DEX incorporation is not advantageous.

### Cell culture experiments

**Fig. 8:**
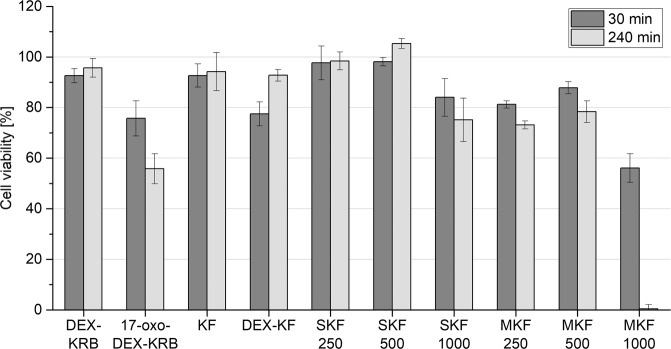
Viability of HCE-T cells, as measured via MTT assay after incubations for 30 and 240 min with DEX-PBS, 17-oxo-DEX-PBS, and various release media from unloaded and DEX-loaded keratin films; the viability after incubation with KRB was set to 100%, mean ± SD, *n* = 6

The effect of sample solutions on metabolic activity and thus on the viability of cells was monitored by MTT assay. The cell viability testing results are displayed in Fig. 8. Incubation of HCE-T cells with saturated DEX-KRB (with a DEX concentration of 69.30 ± 0.28 µg/mL) did not negatively affect the cell viability compared to incubation with KRB (*P* > 0.05). In contrast, the cell viability was reduced after incubation with saturated 17-oxo-DEX-KRB (*P* < 0.05). The 17-oxo-DEX concentration in the sample was determined to be 50.79 ± 0.31 µg/mL. This decrease seemed time dependent, since the cells that were incubated within the sample for 30 min were more viable than the cells that were incubated for 240 min. The viability of HCE-T cells after 240 min of incubation with saturated 17-oxo-DEX-KRB decreased to a minimum of <60%. The viability-reducing effects of DEX-loaded substrates during release were estimated by incubating HCE-T cells with the respective release media. The release medium obtained from an unloaded KF led to no reduction in viability (*P* > 0.05), whereas the medium obtained from directly loaded DEX-KF induced a slight decrease after 30 min of incubation (*P* < 0.05). Since no further adjustments were made to the film composition, the shift must have been influenced by the DEX or 17-oxo-DEX in the films. Considering the effects of saturated KRB solutions, 17-oxo-DEX was likely responsible for this viability reduction. The release media obtained from SKF 250 and SKF 500 caused no decrease in cell viability (*P* > 0.05), although 17-oxo-DEX was also detected in these films during release studies. However, these films proportionally released more DEX than 17-oxo-DEX. Thus, it seems likely that the released DEX antagonized the viability harming impact of 17-oxo-DEX. After incubating the cells within the release medium of SKF 1000 for 240 min, the cells were slightly less viable again (*P* < 0.05). This viability change indicates that there is a limitation for the maximum profitable DEX concentration. More prominent viability reductions were observed after incubation with the MKF release media. While the release media from MKF 250 and MKF 500 led to slightly diminished viabilities after 240 min of incubation (*P* < 0.05), a remarkable level of cell-viability collapse was observed after incubating HCE-T cells with the release medium from SKF 1000 (*P* < 0.05). After an incubation period of 240 min, an almost complete decline (<1%) was observed.

**Fig. 9:**
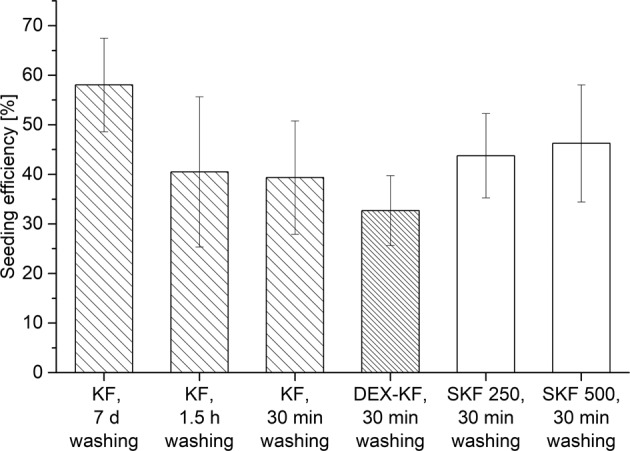
Seeding efficiencies of HCE-T cells on differently washed, unloaded keratin films (KFs) as well as on films fabricated from DEX saturated dialysate (DEX-KFs) and films fabricated from different DEX dialysate suspensions (SKF 250 and SKF 500), mean ± SD, *n* = 4–8

To evaluate the suitability of alternative KF preparation protocols in cell culture experiments, the influences of shortened washing procedures on the seeding efficiencies were determined. This investigation revealed a correlation between reduced washing periods and decreased efficiencies (as displayed in Fig. 9), but none of the efficiencies were found to be significantly reduced (*P* > 0.05). While films that were prepared by the original washing procedure (protocol A) displayed a seeding efficiency of 58%, the efficiency decreased to 41% when the films were washed for only 1.5 h (protocol B). Reducing the washing step even more had no further impact on the efficiency. KFs that were washed for 30 min (protocol C) exhibited a seeding efficiency of 39%. Since a reduced washing protocol is necessary to maintain as much drug content in the films as possible, the 30 min washing approach (protocol C) was confirmed as a standard procedure for further investigations. The preceding experiments with directly loaded KFs revealed satisfactory substrate characteristics, particularly for KFs fabricated from saturated DEX dialysate and for SKFs with low and moderate amounts of suspended drug (SKF 250 and SKF 500). For this reason, these specimens were selected and their potentials were evaluated in contact with corneal epithelial cells. The seeding efficiency on the KFs fabricated from the saturated dialysate mixture was found to be slightly decreased to 33%. On the other hand, SKF 250 and SKF 500 displayed higher efficiencies of 44% and 46%, respectively; this indicated that drug incorporation had no negative impact on the seeding behavior of the examined corneal cell line. In fact, the released DEX in the medium could have even had a positive effect on the cells, as observed during the cell-viability investigation. Nevertheless, all the differences in the seeding efficiencies were not statistically significant (*P* > 0.05).

**Fig. 10:**
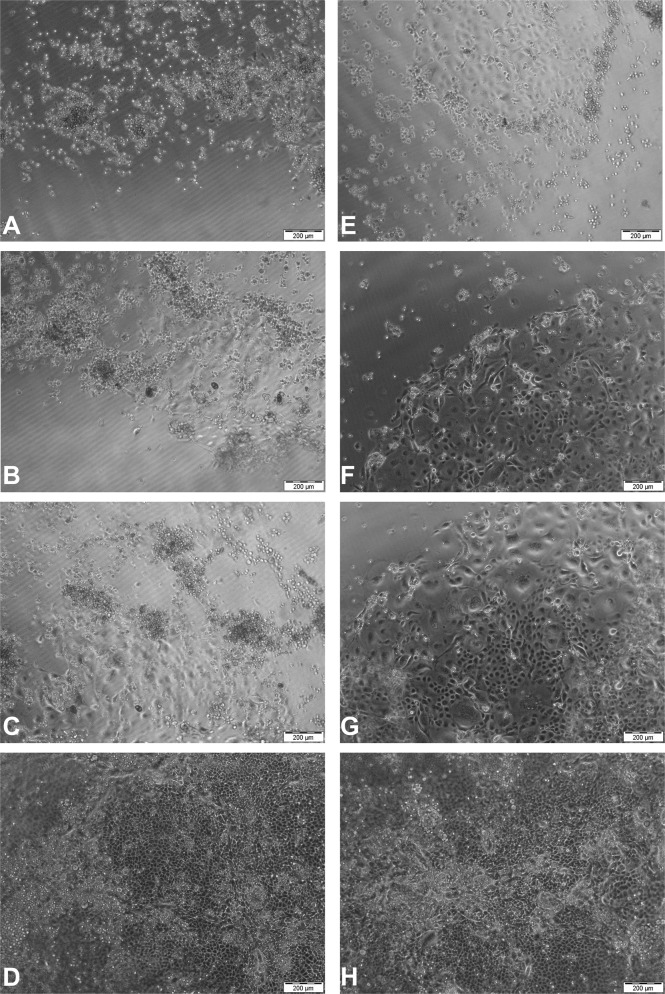
Micrographs of HCE-T proliferation **A** on KF and **E** dir. DEX-KF after 48 h, **B** on KF and **F** dir. DEX-KF after 96 h, **C** on KF and **G** dir. DEX-KF after 168 h, and **D** on KF and **H** dir. DEX-KF after 336 h

While observing cell proliferation on unloaded and directly loaded KFs, an inverse correlation between the amount of incorporated DEX and the lag phase became clear (see Table 1). The more DEX was incorporated in the films, the shorter the lag phase of HCE-T cell proliferation on these films. While the lag phases on KFs and directly loaded DEX-KFs were rather long, at 4.26 and 4.82 days, respectively, the lag phases on SKF 250 and SKF 500 diminished to 1.50 and 0.31 days. On the other hand, the PDT increased significantly on films with incorporated DEX. The PDT of HCE-T cells on KFs was found to be only 2.67 days, whereas PDT on SKF 500 was more than twice as long (6.34 days). Initially, the saturation density of HCE-T cells increased when DEX was incorporated into the films. The saturation density on a DEX-KF almost doubled in comparison to the density on an unloaded KF. When more DEX was added to the films by the suspension technique, a gradual reduction in cell-saturation density was observed. Ultimately, the saturation density on SKF 500 was within the range of the density found on a KF without drug incorporation. These results suggest that DEX has a contradictory effect on the proliferation of corneal epithelial cells; while a low corticosteroid amount seems to be beneficial for cell proliferation, higher amounts of DEX lead to a reduced cell-saturation density. Nevertheless, cell growth was detected on all the examined growth substrates as a matter of principle. Since the transparencies of KFs with suspended DEX were limited, cell-growth monitoring via microscopic observation was only possible on unloaded KFs and DEX-KFs manufactured from saturated DEX dialysate. The resulting micrographs are displayed in Fig. 10. In the beginning, the seeded HCE-T cells on the KF and the DEX-KF had rounded morphologies and were scattered across the film surfaces. The number of cells on films increased noticeably after 4 and 7 days. After this delayed start, cell growth accelerated thereafter. At the end of the period under investigation, the cell monolayers on both substrates were confluent, and the cells exhibited typical epithelial morphologies. These properties indicate successful cell growth on the specimens and lead to the conclusion that the drug-loaded KF is a biocompatible growth substrate.

**Table 1:** Lag phase, population doubling time (PDT) and saturation density based on the proliferation of HCE-T cells on unloaded keratin films (KFs), films from DEX saturated dialysate (DEX-KFs) and films fabricated from different DEX dialysate suspensions (SKF 250 and SKF 500)

	KF	DEX-KF	SKF 250	SKF 500
Lag phase [d]	4.3	4.8	1.5	0.3
PDT [d]	2.7	3.6	4.2	6.3
Saturation density [cells/cm^2^]	173,000	340,000	247,000	151,000

## Discussion

In recent decades, keratin has been established as an interesting material for use in regenerative medicine. Keratin has been proven to support cell adhesion and proliferation and has excellent structural properties since it is an essential part of cornified tissues [13, 14]. However, the strong chemical linkages within these proteins make keratin extraction from natural resources challenging. In previous studies, an optimized formulation procedure was established for maintaining noncytotoxic film structures [16, 17, 28, 31]. Keratin is extracted from human hair by a reductive process called the Shindai method. Afterwards, cytotoxic agents as for example thiourea and 2-mercaptoethanol are removed by dialysis. The high osmolarity of the extract, which adds to its cytotoxic potential, is reduced by this step as well. While dialysis in a neutral pH environment leads to an opalescent, nanoparticulate keratin suspension, dialysis in a fiercely alkaline environment provides a clear dialysate with hydrolyzed protein fragments [16]. For film preparation, dialysates are blended at a ratio of nine parts aqueous fluid to one part alkaline fluid. This ratio is necessary for creating strong films that are sufficiently flexible during the specimen suturing in ocular surface reconstruction. In previous studies, it was determined that films made exclusively from aqueous dialysate were strong but lacked the flexibility to adapt to the ocular surface. On the other hand, films with higher ratios of alkaline dialysate were too fragile to be properly sewed during ocular surface reconstruction [17, 31]. Thus, the clinical applicability of these alternative formulations is mostly limited. Mixing aqueous and alkaline dialysates in suitable proportions produces a liquid with a moderate alkaline pH. However, the pH stability range for corticosteroid formulations, such as DEX, is located on the mildly acidic and neutral spectrum; this makes the direct incorporation of DEX into KFs challenging. Furthermore, it has been acknowledged that DEX degrades in aqueous environments [34]. These oxidative processes are enhanced by elevated temperature or light exposure [34]. 17-oxo-DEX has been reported as a major degradation product of oxidation under basic stress conditions [35–37]. Water is eliminated from DEX after the C_20_ keto group is converted into an enol group [36, 38]. We also observed considerable degradation of the incorporated DEX after curing. The degradation product could be identified as 17-oxo-DEX by comparison to an external standard. To the best of our knowledge, the effect of 17-oxo-DEX on ocular tissues has not yet been described. However, it is essential to cure KFs to induce strong and water-insoluble structures since disulfide linkages within keratin are strengthened by the reoxidation of cysteine residues during this step [16]. Thus, a reduction in the thermal energy input is not possible. In vitro release profiles show that DEX degradation increases with the amount of solved DEX in the films. When increasing the incorporated DEX amount by the suspension technique, on the other hand, this decomposition is more limited. In addition, extended API release is ensured by the slow dissolution of the homogenously dispersed crystals. This result indicates that the suspension of DEX within these films is the most promising approach for drug incorporation. On the other hand, no degradation was found after subsequent incorporation of DEX. Nevertheless, rapid API release from the KF after subsequent incorporation contradicts the clinical benefit of this approach.

Considering the artificial setting of the conducted in vitro release experiments, data must be discussed carefully. Ocular bioavailability in vivo is strictly limited by the given physical conditions. Approximately only 3% of a topically applied drug is found in the aqueous humor after instillation [19]. In vivo tear fluid dynamics cause continuous dilution and rapid removal of the administered API from the ocular surface. Furthermore, the volume of in vivo tear fluid is much smaller than the capacity of the release medium in vitro. For this reason, in vitro data from static experiments can provide only a comparative view and implications about the prolonged release characteristics. In this way, the most promising substrates can be selected for further experiments. The exact concentration of DEX, which is needed to prevent inflammation after surgery, has not yet been investigated, but it was estimated previously that a concentration of 1 µg/mL can accomplish anti-inflammatory efficacy [19, 39]. Therefore, the obtained release profiles of SKFs are promising for supplying suitable concentrations of API over an extended period. While MKFs provided these DEX concentrations over only 3 days to 1 week, SKFs delivered satisfactory concentrations over more than 1 week or even within the entire investigation period. Nonetheless, more in vivo-like experiments must be performed to prove that these films exhibit effective anti-inflammatory activities.

For ocular surface reconstruction suitability, a material must have some essential characteristics. In particular, good transparency, adequate mechanical strength and excellent biocompatibility are of great importance in clinical applicability [40]. In previous studies, KFs have been proven to fulfil all these criteria [16, 17]. It was shown that KFs are, at least, not inferior to the amnion membrane, which is the current treatment option of choice. KFs displayed higher transmission levels compared to amnion membranes, and their biomechanical properties were within the same range as those reported for amnion membranes [16]. The KF ultrastructure is crucial for these beneficial traits. KFs are made up of numerous tightly packed keratin nanoparticles, which are found in aqueous dialysate [16, 28]. After drying, the nanoparticles are closely connected to one another, and the final curing step accomplishes further nanostructure linkages by disulfide bonds [16]. In the current study, we determined that drug incorporation is a threat to the maintenance of this ultrastructure and to the positive film characteristics.

Films that were fabricated from saturated dialysate demonstrate high transparencies, which are comparable to the light-transmission properties of contact lenses. The high transparency of a KF is based on the repetitive assembly of spherical nanoparticles from aqueous dialysate and the absence of a superior, filamentous structure [16]. Since we did not detect recrystallization of DEX in the films, we assume that the compounds are molecularly dispersed within the dense arrangement of particles and thus do not affect the optical characteristics of the films. Films with suspended DEX allow less light to pass and therefore appear opalescent; this characteristic is compounded when more substance is suspended in the films. In a previous study, we found the keratin nanoparticle diameter to be 120 nm with a rather narrow particle size distribution [16]. However, we microscopically observed that the DEX crystals in these films have particle sizes in the smaller micrometer range; thus, they are much larger than keratin nanoparticles and consequently interfere with the tightly packed nanoparticle order. Therefore, light is prevented from directly passing through these films and is bent or reflected by the DEX crystals instead. KFs with solubilizing agents display limited transparencies, although no DEX crystals were observed microscopically. This result can be explained by the superordinate structures of PS80 and P40S, which assemble during temperature exposure. These areal structures interrupt the proper nanoparticle order of plain films even more than the relatively homogenous dispersed, micronized drug crystals observed in SKFs. Consequently, the transparency is observed to further decrease. Nevertheless, compared to AM, the high light-transmission potentials of DEX-KF, SKF 250 and SKF 500 seem satisfactory and represent an improvement.

The surgical procedure of ocular surface reconstruction must be performed with hydrated films. Thus, evaluations of the E-modulus and ultimate strength were conducted with films that were equilibrated in water. It was reported previously that the biomechanical strength of hydrated specimens weakened due to the reduced stability of particle interactions [16]. We observed a further reduction in the US upon drug incorporation. In accordance with these findings, only slight changes were detected in films fabricated from saturated dialysate. Since we assume that DEX is molecularly dispersed within the films, the distance between keratin nanoparticles is only marginally affected by DEX. A different situation arises when DEX is incorporated by the suspension technique. The distances between keratin particles greatly increase at locations where drug crystals are embedded in the grid, and the interparticular bonds are therefore weakened; as a consequence, we observed reduced US values. The existing results for KFs with solubilizing agents are consistent with this theory. The merged structures of PS80 and P40S interrupt keratin nanoparticle interactions and thus are responsible for these decreased US values. The evaluation of KF mechanical properties after subsequent DEX incorporation displayed decreased US values. Similar behavior was observed after KF incubation in PBS without DEX. This result indicates that the interparticular forces within the KF are weakened by the PBS solution salts upon loading.

In addition to biomechanical testing, the swelling behavior of the films was assessed. Previous studies offered extensive insight into the biomechanical and swelling characteristics of film specimens with varying proportions of alkaline dialysate. It was shown that alkaline dialysate extensively affected the film demeanor during equilibration in water [16]. Thus, it was investigated whether drug incorporation had a similarly strong influence on swelling and whether this might permit a correlation to biomechanical properties. The results from swelling studies indicated no such effect, since the swelling behaviors of all the films were comparable. Merely, the films with incorporated solubilizing agents exhibited diversified behaviors during equilibration; this is most likely due to the high share of amphiphilic molecules within the specimens, but the exact reasons have not been conclusively clarified. However, it must be pointed out, that swelling study was conducted using a one-dimensional approach. Only the increase of KF diameter was investigated. Thus, no conclusion concerning anisotropies of the material can be drawn. Evaluation of swelling using a gravimetric method was not feasible, since incorporated molecules were washed out of the specimens during the swelling process and distorted the gravimetric data.

To analyze the biocompatibility, HCE-T cells were seeded onto the films. Previous studies have shown that keratin contributes to cell adhesion and proliferation. In particular, fibroblasts were subjected to these investigations [41–43]. We also proved that HCE-T cells proliferate on KFs. In a previous study it was shown that in comparison to AM, the seeding efficiency slightly decreased, and the length of the lag phase marginally increased on KFs. Nevertheless, the supportive effects of KF on cells were quite sufficient [16]. In the present study, we demonstrate that KF preparation for cell culture can be abbreviated and a shortened washing protocol is established. The cells that were seeded on the films that were treated under these simplified conditions showed a reduced but still satisfactory seeding efficiency. KFs with suspended DEX supported seeding as well. While films fabricated from saturated DEX dialysate were found to support seeding slightly less, films with increasing amounts of suspended DEX were characterized to have a higher number of attached cells after the investigation period. This higher number of attached cells could suggest that the seeding efficiency is promoted by corticosteroids. Since corticosteroids are frequently added to cell culture media as differentiation supplements, this result seems consistent with current practices [22, 44, 45]. Either way, the high drug concentrations within the films and the released DEX and 17-oxo-DEX do not seem to limit cell attachment, although corticosteroids have been shown to promote cell apoptosis [22, 46, 47]. During cell-viability testing, we observed no decrease in metabolic activity after HCE-T cells were incubated with saturated DEX solution. On the other hand, at saturation concentrations, 17-oxo-DEX showed a viability-reducing effect on HCE-T cells during the MTT assay. Nevertheless, this effect did not translate into similar reductions in the seeding efficiencies on substrates containing higher amounts of the DEX degradation product.

The biocompatibility was further assessed by investigating the possible viability-reducing effects of DEX-loaded KFs during release. There was a remarkable decline in cell viability after HCE-T cells were incubated with the MKF release media; this might partly be explained by the substantial proportion of DEX degradation products within these films. Considering the fact that the DEX in MKF degrades into 17-oxo-DEX on a large scale, the viability-reducing influence of 17-oxo-DEX might be responsible for the decline observed after incubation with the release media from MKF. Another aspect of this behavior might be caused by the high shares of amphiphilic substances in MKF substrates, even though no irritation was induced by the mixed nanomicelles from PS80 and P40S in an in vivo rabbit model [32]. Nonionic surfactants such as PS80 have shown relatively low potentials for cytotoxicity and irritation [48–50], but high concentrations of these molecules might adversely affect the integrity of the cell membrane and cell equilibrium to a certain extent. Since no viability reduction was found after incubation with SKF release media, the suspension approach once again seems to be the method of choice for direct drug incorporation into KFs.

An investigation of cell proliferation on the film substrates indicates a more diversified situation than that characterized with seeding efficiency testing. In accordance with the literature, we found that DEX had an ambivalent impact on corneal epithelial cells during growth [22]. While small amounts of DEX proved to be beneficial for proliferation, higher shares of the incorporated drug restricted cell growth and led to reduced cell-saturation densities. This result might be explained by rapidly induced cell differentiation, which is also evident from the rather short lag phases on the specimens with higher amounts of incorporated DEX. In practice, films with lower amounts of incorporated DEX could be of particular interest for generating clinically beneficial inflammation suppression after surgery. Nevertheless, this theory must be confirmed under more in vivo-like conditions. In particular, the growth potential of limbal stem cells on substrates might be an interesting topic for further investigation. A balance between maintaining positive substrate characteristics and supplying adequate amounts of DEX to achieve a considerable anti-inflammatory effect at the transplant site must be retained.

## Conclusion

In this study, drug-loaded KFs were successfully established. Regarding chemical stability and prolonged release, the suspension technique was identified as the most suitable method for the direct incorporation of DEX. The amount of incorporated DEX had a strong influence on the transparency and biomechanical strength of the films. Nevertheless, even upon the incorporation of a high DEX quantity, the film properties were still within an acceptable range. Moreover, cell culture studies indicated that the drug load had no negative effect on biocompatibility. It can be concluded that the KF with a small amount of suspended DEX is an interesting material that is suitable for replacing the AM used in ocular surface surgery. The anti-inflammatory component of these films could be clinically beneficial and prevent extensive inflammation at the transplant site after surgery. The suitability of sterilization procedures for the drug-loaded specimens, the transferability of in vitro data to the in vivo situation and the evaluation of the clinical applicability of these drug-loaded films are subjects that must be investigated in the future.

## Supplementary Information

Supplementary Material Fig. S1

Supplementary Material Fig. S2

Supplementary Material Fig. S3

Figure Legends
