# Supplementary figures and images for: Dexamethasone-loaded keratin films for ocular surface reconstruction
Source: J Mater Sci Mater Med. 2022 Mar 4;33(3):29. doi: 10.1007/s10856-021-06638-z (PMC9050765; doi:10.1007/s10856-021-06638-z)

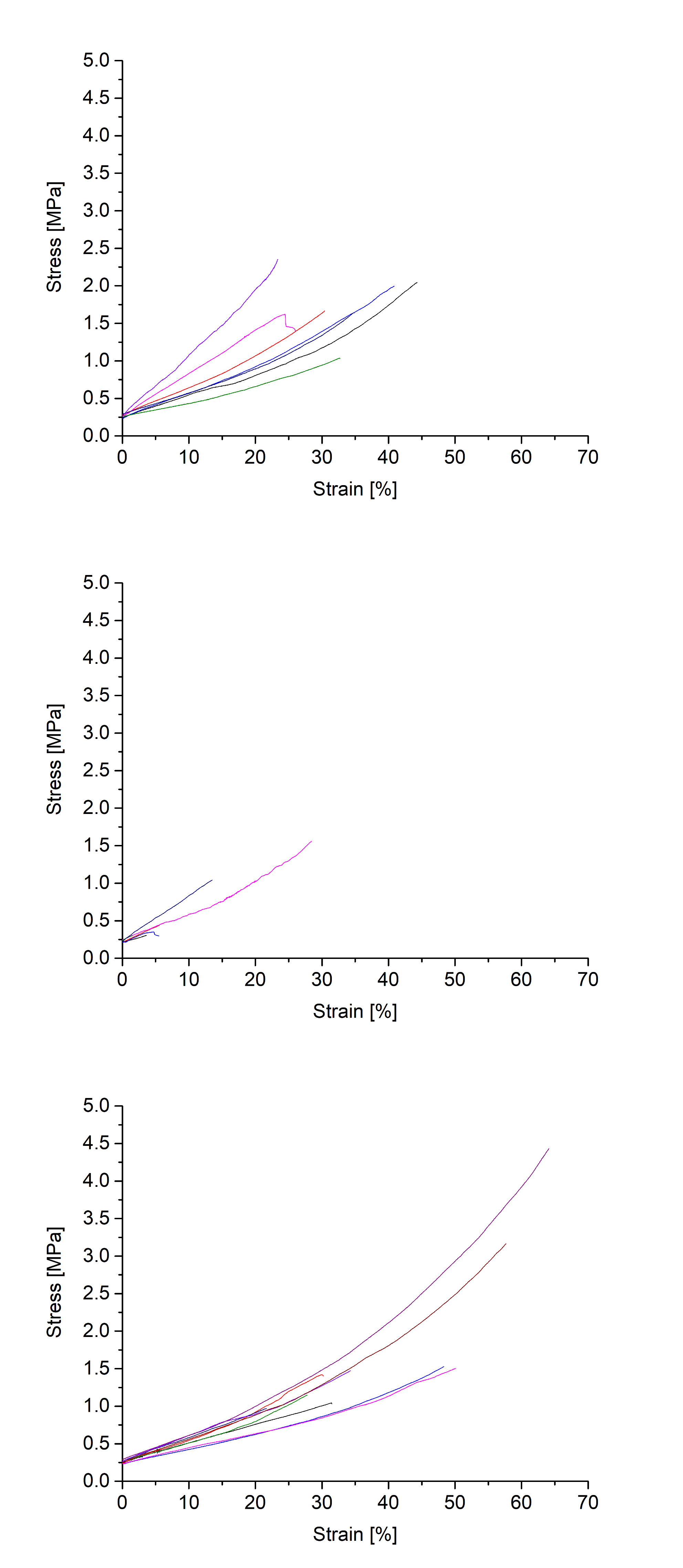

Supplement: Supplementary file 1 — Supplementary Material Fig. S1 [file 10856_2021_6638_MOESM1_ESM.tif]

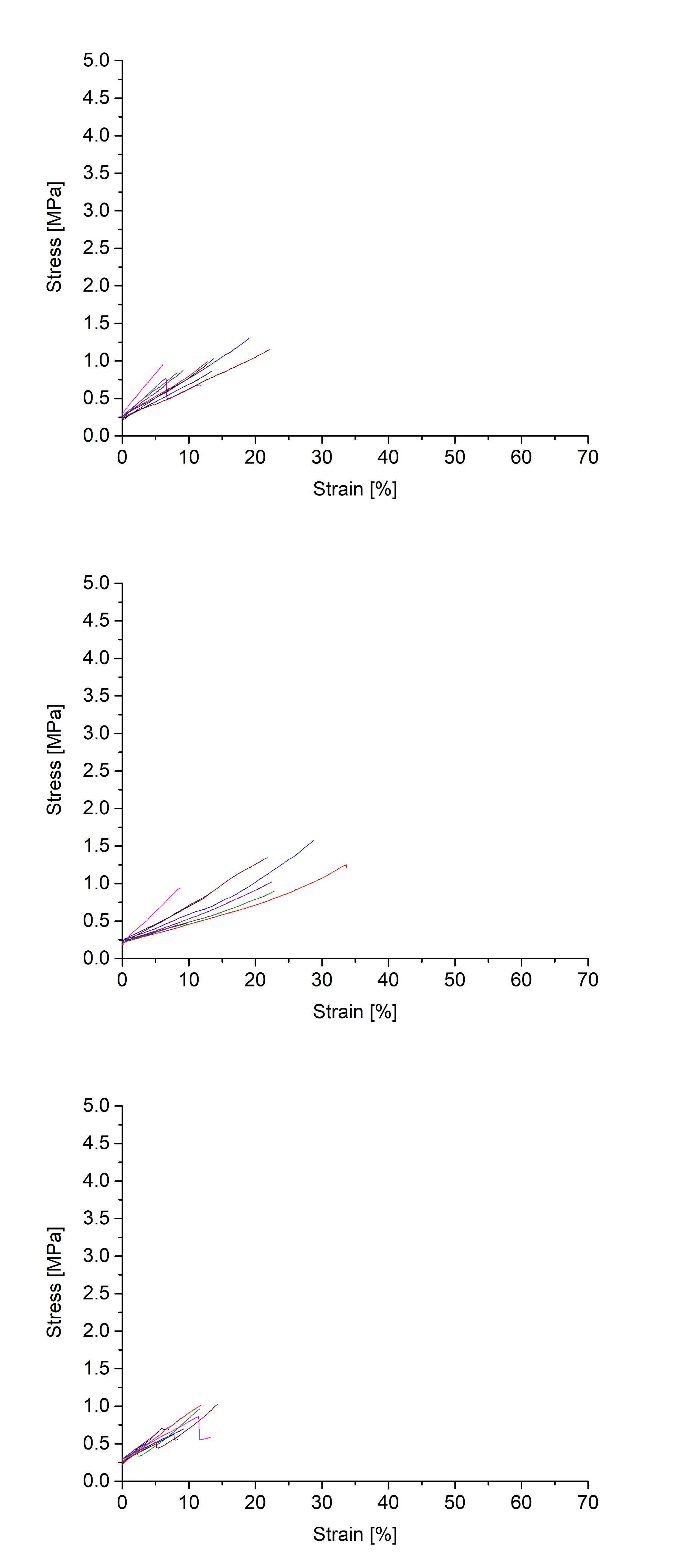

Supplement: Supplementary file 2 — Supplementary Material Fig. S2 [file 10856_2021_6638_MOESM2_ESM.tif]

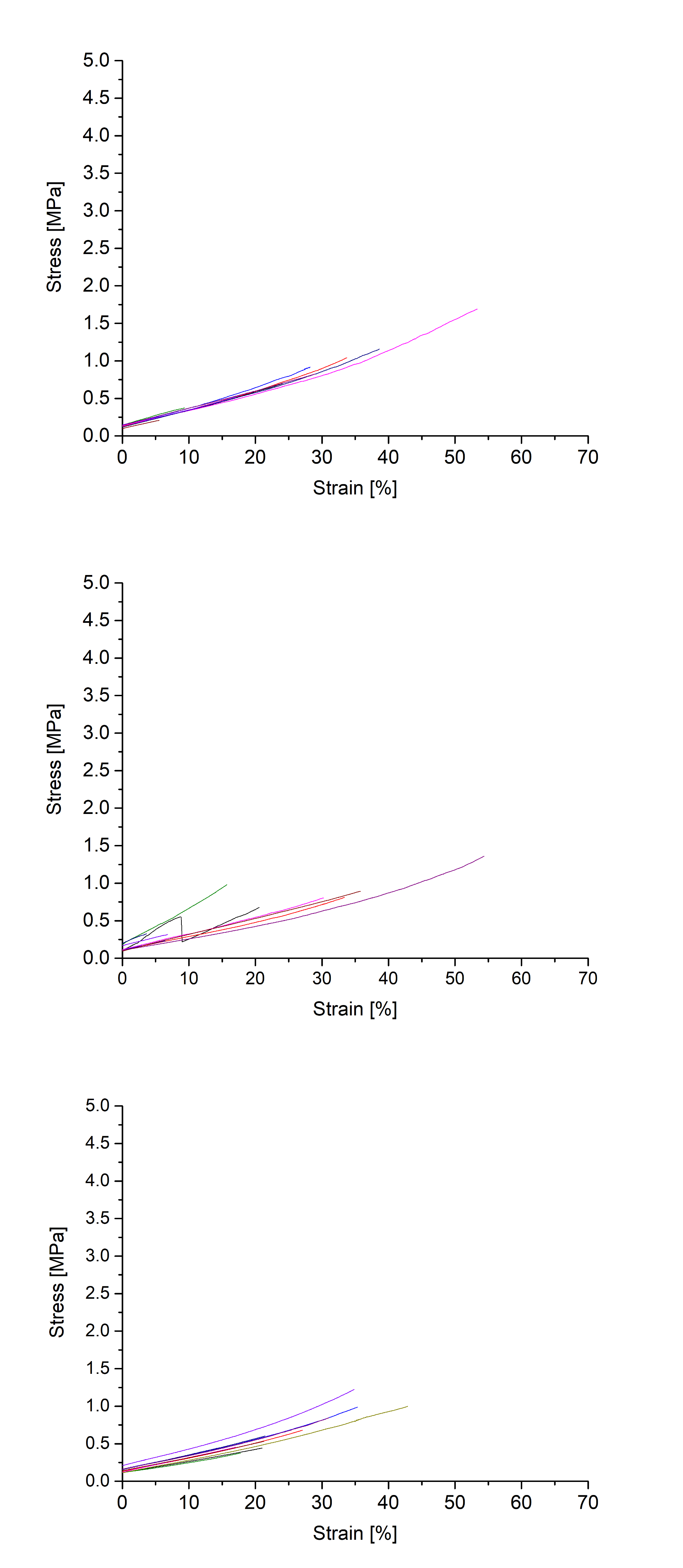

Supplement: Supplementary file 3 — Supplementary Material Fig. S3 [file 10856_2021_6638_MOESM3_ESM.tif]
