# Supplementary material for: Dexamethasone-loaded keratin films for ocular surface reconstruction
Source: J Mater Sci Mater Med. 2022 Mar 4;33(3):29. doi: 10.1007/s10856-021-06638-z (PMC9050765; doi:10.1007/s10856-021-06638-z)
Supplement: Supplementary file 4 — Figure Legends [file 10856_2021_6638_MOESM4_ESM.pdf]

Dexamethasone-loaded keratin films for ocular surface reconstruction

Rebekka Schwab, Stephan Reichl

Supplementary Material

First figure

Resulting stress-strain-curves from mechanical testing of unloaded keratin films (top) as well as for subsequently loaded films (middle) and films fabricated from DEX saturated dialysate (bottom), n = 6-9

Second figure

Resulting stress-strain-curves from mechanical testing of films fabricated from different DEX dialysate suspensions: SKF 250 (top), SKF 500 (middle) and SKF 1000 (bottom), n = 9-10

Third figure

Resulting stress-strain-curves from mechanical testing of films manufactured with different amounts of solubilized DEX: MKF 250 (top), MKF 500 (middle) and MKF 1000 (bottom), n = 9-10
